# 

**Published:** 1903-12

**Authors:** 


### The Library of the Bristol Medico-Chirurgical Society.

*The following donations have been received since the publication of the List in September :*

*November 30th, 1903.*

| | | |
|---|---|---|
| Cassell and Company, Ltd. (1)... ... ... ... ... ... | 1 | volume. |
| The Manager of *The Colliery Guardian* (2) ... ... | 1 | ,, |
| Hubert Kerslake (3) ... ... ... ... ... ... | 1 | ,, |
| The Royal College of Physicians of London (4)... | 1 | ,, |
| R. Shingleton Smith, M.D. (5)... ... ... ... ... | 5 | volumes. |
| The Society for the Study of Disease in Children (6) | 1 | volume. |
| The Surgeon-General, United States Army (7) ... | 1 | ,, |
| University College, Bristol (8) ... ... ... ... ... | 1 | ,, |

Pamphlets have been received from Mr. L. M. Griffiths and Dr. R. Shingleton Smith. Some unframed portraits have been presented by Mr. L. M. Griffiths.

### FIFTIETH LIST OF BOOKS.

The titles of books mentioned in previous lists are not repeated.

The figures in brackets refer to the figures after the names of the donors, and show by whom the volumes were presented. The books to which no such figures are attached have either been bought from the Library Fund or received through the *Journal.*

**Allchin, W. H.** [Ed.] *A Manual of Medicine.* Vol. V. ... ... ... ... ... 1903

*Ankylostomiasis : its Cause, Treatment, and Prevention* ... ... ... (2) 1903

*Atlas of Illustrations of Clinical Medicine, Surgery, and Pathology, An* N. S. Fasc. I.–VII. 1902–03

**Atlay, J. B.** ... ... *Sir Henry Wentworth Acland, Bart : A Memoir.* 1903

**Beale, P. T. B.** ... ... *Aids to Physiology* ... ... ... ... ... ... ... ... 1903

**Bishop, E. S.** ... ... *Essentials of Pelvic Diagnosis* ... ... ... ... ... ... 1903

**Bramwell, J. M.** ... *Hypnotism* ... ... ... ... ... ... ... ... ... ... ... 1903

**Carpenter, G.** ... ... *Golden Rules for Diseases of Children* ... 2nd Ed. [1903]

**Carter, R. B.** ... ... *Doctors and their Work* ... ... ... ... ... ... ... 1903

*Catalogue (Index) of the Library of the Surgeon-General's Office, United States Army* ... ... ... ... ... ... (7) 2nd Ser. Vol. VIII. 1903

*Catalogue of Accessions to the Library of the Royal College of Physicians of London during 1903* ... ... ... ... ... ... ... ... ... ... (4) 1903

**Chapman, T. A.** ... *Medico-Psychological Association of Great Britain and Ireland. A Revision of the Statistics presented by the Committee on Tuberculosis* ... ... 1903

**Coats, J.** ... ... ... *Manual of Pathology.* (Ed. by L. R. Sutherland.) 5th Ed. 1903

**Cunningham, D. J.** ... *Manual of Practical Anatomy.* Vol. II. 3rd Ed. 1903
**Edgar, J. C.** ... ... *The Practice of Obstetrics* ... ... ... ... ... ... ... 1903
**Ellison, Mary A.** ... *Manual of Massage.* (Revised by G. Manley.) 2nd Ed. 1904 (*sic*)
**Fink, G. H.** ... ... *Cancer and Precancerous Changes* ... ... ... ... ... 1903
**Fothergill, W. E.** ... *Manual of Midwifery* ... ... ... ... 3rd Ed. 1903
**Gould, A. P.** ... ... *Elements of Surgical Diagnosis* ... (1) 3rd Ed. 1903
**Hall, F. de H.** ... ... *The Medical Examination for Life Assurance* 3rd Ed. 1903
**Harley, G.** ... ... *Diseases of the Liver* ... ... ... ... ... ... (5) 1883
**Hayden, T.** ... ... *Diseases of the Heart and Aorta* ... ... ... (5) 1875
**Heine, B.** ... ... ... *Operationen am Ohr.* ... ... ... ... ... ... 1904 (*sic*)
**Heinricius, G.** ... ... *Barnbördshusets och den Gynäkologiska Klinikens i Helsingfors* ... ... ... ... ... ... ... 1902
**Hunt, W. H.** ... ... *Illustrated Ventnor* ... ... ... ... ... ... (5) 1903
**Jardine, R.** ... ... *Practical Text-Book of Midwifery* ... ... 2nd Ed. 1903
**Jones, A. H. Tubby and R.** *Surgery of Paralyses* ... ... ... ... ... ... 1903
**Lake, R.** ... ... ... *Diseases of the Ear* ... ... ... ... ... ... ... 1903
**Lewers, A. H. N.** ... *Diseases of Women* ... ... ... ... ... 6th Ed. 1903
**Lysons, Rev. D.** ... *A Sketch of the Life and Character of Charles Brandon Tyre, F.R.S.* ... ... ... ... (3) 1812
**See, G.** ... ... ... *De la Phtisie bacillaire des Poumons* ... ... (5) 1884
**Sheild, A. M.** ... ... *Surgical Lectures and Essays* ... ... ... ... ... ... [N.D.]
**Smith, W. J.** ... ... *Surgical Bandaging* ... ... ... ... ... ... ... [N.D.]
**Swanzy, H. R.** ... *Handbook of Diseases of the Eye* ... ... 8th Ed. 1903
**Tubby and R. Jones, A. H.** *Surgery of Paralyses* ... ... ... ... ... 1903
**Vincent, R.** ... ... *The Nutrition of the Infant* ... ... ... ... 1904 (*sic*)
**Watson, J. K.** ... ... *The Examination of the Urine* ... ... ... ... ... [N.D.]
*Where shall I send my Patient?* ... ... ... ... ... ... ... ... ... ... ... 1903

### TRANSACTIONS, REPORTS, JOURNALS, &c.

American Gynecology ... ... ... ... ... ... ... ... ... Vol. II. 1903
American Journal of Obstetrics, The ... ... ... ... Vol. XLVII. 1903
American Laryngological Association, Transactions of the ... ... ... 1903
American Medicine ... ... ... ... ... ... ... ... ... ... Vol. V. 1903
American Ophthalmological Society, Transactions of the Vol. X., Pt. I. 190
American Pediatric Society, Transactions of the ... ... Vol. XIV. 1902
Boston Medical and Surgical Journal, The... ... ... Vol. CXLVIII. 1903
British Gynæcological Journal, The ... ... ... ... ... Vol. XVIII. 1902
British Medical Journal, The ... ... ... ... ... ... Vol. I. for 1903
Buffalo Medical Journal ... ... ... ... ... ... ... ... Vol. XLII. 1903
Calendar of University College, Bristol, for 1903-04 ... ... ... (8) 1903
Cincinnati Lancet-Clinic, The ... ... ... ... ... Vol. LXXXIX 1903
Clinical Journal, The ... ... ... ... ... ... ... ... Vol. XXI. 1903
Clinical Society's Transactions ... ... ... ... ... Vol. XXXVI. 1903
Congrès international de Médecine professionelle et de Déontologie médicale, Comptes Rendus de 1ere Session ... ... (5) 1900
Edinburgh Obstetrical Transactions ... ... ... ... Vol. XXVIII. 1903
Glasgow Medical Journal, The ... ... ... ... ... Vol. LIX. 1903
Hospital, The ... ... ... ... ... ... ... ... ... ... Vol. XXXIV. 1903

Hospitals—
Westminster Hospital Reports, The ... ... ... Vol. XIII. 1903
Journal of Laryngology, Rhinology, and Otology, The ... Vol. XVII. 1902
Journal of Medical Research, The ... ... ... .. ... ... Vol. IX. 1903
Journal of Obstetrics and Gynæcology of the British Empire, The Vol. III. 1903
Journal of the American Medical Association, The ... ... ... Vol. XL. 1903
Lancet, The ... ... ... ... ... ... ... ... ... ... Vol. I. for 1903
Liverpool Medico-Chirurgical Journal, The ... ... ... Vol. XXII. 1902
Medical News, The ... ... ... ... ... ... ... ... ... Vol. LXXXII. 1903
Medical Press and Circular, The ... ... ... ... [Vol. CXXVI.] 1903
Medical Record . ... ... ... ... ... ... ... ... ... ... Vol. LXIII. 1903
Medico-Chirurgical Transactions ... ... ... ... ... Vol. LXXXVI. 1903
New York Medical Journal, The ... ... ... ... ... Vol. LXXVII. 1903
New York, Transactions of the Medical Society of the State of ... ... 1903
Practitioner, The ... ... ... ... ... ... ... ... ... [Vol. LXX.] 1903
Quarterly Medical Journal, The ... ... ... ... ... ... Vol. XI. 1902-03
Scottish Medical and Surgical Journal, The ... ... .. Vol. XII. 1903
Society for the Study of Disease in Children, Reports of the (6) Vol. III. 1902-03
Year-Book of Scientific and Learned Societies... ... ... ... ... ... 1903

### MEETINGS OF SOCIETIES.

### Bristol Medico-Chirurgical Society.

### Local Medical Notes.

**University College, Bristol.**—Prof. Sydney Young has been appointed to the Chair of Chemistry, Trinity College, Dublin. Dr. F. J. Poynton, a former student of this College, has been appointed Assistant-Physician to University College Hospital, London. The following students were successful in the recent M.B. Examination of the University of London, viz.:—*1st Division*, A. R. Short, B.Sc.; *2nd Division*, J. J. S. Lucas, B.A., M.R.C.S., J. D. C. Calcott, J. E. Sparks, M.R.C.S., A. W. C. Richardson. The following students have obtained the M.R.C.S. and L.R.C.P. Diplomas:—F. H. Pickin, R. H. N. Rutherford, and W. T. Webb.

**Bristol Royal Infirmary.**—J. A. Nixon, B.A., M.B., B.C. (Cantab), has obtained the M.R.C.P. (Lond.) Diploma. The following appointments have been made, viz.:—Assistant House Physician and Resident Obstetric Officer, N. A. W. Conolly, M.R.C.S., L.R.C.P.; Assistant House Surgeon and Anæsthetist, W. T. Webb, M.R.C.S., L.R.C.P.; Casualty Officer, C. W. W. James, M.R.C.S., L.R.C.P.

**Bristol General Hospital.**—The following appointments have been made:—Assistant House Physician, H. Devine, M.R.C.S., L.R.C.P.; Assistant House Surgeon, F. H. Pickin, M.R.C.S., L.R.C.P.; Assistant House Surgeon in the Casualty Department, C. A. Moore, M.R.C.S., L.R.C.P.

**Bristol Dispensary.**—Messrs. Charles Corfield, M.R.C.S., L.R.C.P., and Leonard A. Moore, M.R.C.S., L.R.C.P., have been appointed Medical Officers to this Institution.

**Bristol Eye Dispensary.**—The new premises recently added to the Orchard Street Eye Dispensary were formally opened on Nov. 10th by Miss Tyndall, under the presidency of Mr. P. J. Worsley, who gave the following interesting account of the history of this useful Institution. He remarked that, as that was almost the first occasion upon which there had been anything like a meeting of the public at that Dispensary, it might be interesting to hear shortly what the origin of the Institution was. He presumed that there must have been a very considerable need of eye dispensaries and hospitals in Bristol about the beginning of the last century, because the Eye Hospital and that Dispensary were founded within two years of one another —in 1810 and 1812 respectively. What Bristol did in this respect before that he did not know, and he was afraid the poor people must have had rather a bad time. About 1812 Mr. Estlin, a well-known surgeon, was living in Park Street, and began, he believed, seeing poor patients at his own house gratuitously. He went on with that for some forty years—a very long time for a practising surgeon to be able to continue a work of that nature. Mr. Estlin had a nephew—Mr. Augustin Prichard—who was of the same surgical ability, and was for a very long time the oculist of Bristol. He succeeded his uncle, and carried on the Dispensary, not, he thought, in Orchard Street, but somewhere in the neighbourhood. His friends helped him to extend the scope of the Dispensary, money being found to take a house for it, and Mr. Prichard worked at it himself for no less than sixty-two years, which was, he thought, almost an unparalleled record. He managed it with great economy, and it was perfectly marvellous what an amount of good work he was able to do upon the slender means furnished to him. He received a few bequests and gifts, which he husbanded with very great care and skill. He wished the Dispensary to become more of a public institution, and he got together a committee, to whom he handed over the little funds he had accumulated, together with the lease of the old house in Orchard Street. He was assisted for some time by Mr. Crosby Leonard. Fortunately, Mr. Prichard had a son, and when he retired Mr. Arthur Prichard was able to take up the work. He associated with himself Mr. Cyril Walker and Dr. Ogilvy. Such, briefly, was the history of the Institution. It was the effort of one family, and work of which a family might well be proud, and they might be proud to have families in Bristol who were doing such good work.

### INDEX.

(R.) = Review.

Acland, Sir Henry Wentworth—J. B. Atlay (R.), 361.
Allchin, Dr. W. H. [Ed.]—A manual of medicine (R.), 60.
Allingham, H.W.—Operative surgery (R.), 265.
Ambulance in civil life on land and sea, The—R. Harrison (R.), 161.
American Dermatological Association, Transactions of the (R.), 273.
American Public Health Association, Reports of the (R.), 366.
Anæsthetics—Dr. J. Blumfeld (R.), 164.
Anatomy, Heath's practical (R.), 65.
Anatomy, Regional—Dr. R. J. A. Berry (R.), 163.
Anatomy, Surface—Dr. B. C. A. Windle (R.), 163.
Antenatal pathology and hygiene, Manual of—Dr. J. W. Ballantyne (R.), 57.
Armstrong and J. E. Harburn, W. —Buxton: its waters, &c. (R.), 368.
Ascites, Surgical treatment of, 150
Assimilation, digestion, &c., Disorders of—Sir L. Brunton (R.), 263.
Association of American Physicians, Transactions of the (R.), 273.
Atlay, J. B.—Sir Henry Wentworth Acland: a memoir (R.), 361.
Auricles on the percussion of the heart, The influence of the—Dr. D. R. Paterson, 110.

Baby, Our—Mrs. J. L. Hewer (R.), 271.
Bacteriology, Manual of—Drs. R. Muir and J. Ritchie (R.), 70.
Ballantyne, Dr. J. W.—Manual of antenatal pathology and hygiene (R.), 57.
Banti's disease, A case of—Dr. J. M. Clarke, 14.
Barclay, Obituary notice of Wilfred Martin, 187.
Bath, Medical guide to the hot mineral baths of (R.), 65.
Berry, Dr. R. J. A.—Regional anatomy (R.), 163.
Biographic clinics—Dr. G. M. Gould (R.), 358.
Blood, Diseases of the—Dr. A. C. Coles (R.), 69.
Blood, The biological test for, 248.
Blood-counts, The practical value of —Dr. W. Gordon, 234.
Blood-pressure, 144.
Blumfeld, Dr. J.—Anæsthetics (R.), 164.
Brain tumours, Surgical treatment of, 348.
Bridge, Dr. N.—Tuberculosis (R.), 360.
Bristol Medico-Chirurgical Society, 83, 179, 379.
Bristol's awakening, 73.
British Medical Association, Swansea meeting of the, 279.
Bronchi, On rhythmical contractions of the—Dr. P. W. Williams, 6.
Brunton, Sir L.—On disorders of assimilation, digestion, &c. (R.), 263.
Bush, J. P.—Some surprises in my professional career, 289
Bushnell, Dr. F.—The antibodies in disease, 221.
Buxton—W. Armstrong and J. E. Harburn (R.), 368.

Calvert, Dr. J.—Practical pharmacy and prescribing (R.), 366.
Cancer and other tumours of the stomach—Drs. S. and W. S. Fenwick (R.), 263.
Cape Coast Town, Report of the sanitary conditions of—Dr. M. L. Taylor (R.), 70.
Carwardine, T.—Report on surgery, 252; The dangers of delay in ovariotomy, 309.
Chadwick, Obituary notice of Peter Chester, 285.
Chapman, Dr. T. A.—Tuberculosis statistics presented to the Medico-Psychological Association (R.), 364.
Chiene, Professor J.—Mobility, 97,

Clarke, Dr. J. M.—A case of Banti's disease, with autopsy, 14; Report on medicine, 345.
Clinical essays and lectures—H. Marsh (R.), 57.
Coles, Dr. A. C.—The blood: how to examine and diagnose its diseases (R.), 69.
Colitis, muco-membranous—Dr. Froussard (R.), 275.
Collargol—Dr. J. M. Fortescue-Brickdale, 337.
Colman, Obituary notice of Thomas John, 94.
Consumption—Pulmonary consumption—Dr. A. Latham (R.), 66; Notification of phthisis, 76.
Contrexéville, Thirty-five years at—Debout D'Estrées (R.), 367.
Corfield, Dr. W. H.—The etiology of typhoid fever and its prevention (R.), 366.
Crouch, Dr. C. P.—A granuloma of the nose, due to iodide of potassium, 231.

Debout D'Estrées—Thirty-five years at Contrexéville (R.), 367.
Dental anatomy and physiology, Aids to—A. S. Underwood (R.), 164.
Digestive glands, The work of the—J. P. Pawlow (R.), 158.
Disease, The antibodies in—Dr. F. Bushnell, 221.
Duodenal ulcer, A case of perforated—G. M. Smith, 218.

Ear, Diseases of—Dr. H. Macnaughton-Jones [*et al.*] (R.), 59.
Eclampsia, Puerperal, 153
Edgeworth, Dr. F. H.—On some of the initial signs of pleurisy, 36.
Edinburgh medical journal (R.), 68.
Editorial notes, 71, 165, 278, 369.
Ekgren, Dr. E.—Tasenbuch der massage (R.), 69.
Electro-therapeutic work in medicine and surgery, 74, 256.
Electro-therapeutics, Report on—J. Taylor, 256.
Elliott, Dr. C.—A case of infantile scurvy or scurvy-rickets, 323.
Encyclopædia medica (R.), 260.
Epidemiological Society, Commemoration volume of the (R.), 273.

Face, mouth, and jaws, Surgical diseases of the—Dr. H. Grant (R.), 266.
Facial paralysis, The operative treatment of, 255.
Fenwick, Drs. S. and W. S.—Cancer and other tumours of the stomach (R.), 263.
Finsen light treatment. *See* Light treatment.
Firth, Dr. J. L.—Report on surgery, 48.
Fisher, Dr. T.—Report on medicine, 44.
Foods and drugs, Suggested standards of purity for—C. G. Moor (R.), 59.
Forensic medicine and toxicology—Dr. J. D. Mann (R.), 64; Drs. F. Peterson and W. S. Haines (R.), 267.
Fortescue-Brickdale, Dr. J. M.—Collargol, 337.
Fractures and dislocations, Atlas and epitome of—Prof. Helferich (R.), 368.
Fractures, The treatment of—Dr. C. L. Scudder (R.), 276.
Froussard, Dr.—Muco-membranous colitis (R.), 275.
Fry, Obituary notice of Albert, 286.

Giles, Dr. A. E.—Menstruation and its disorders (R.), 62
Gordon, Dr. W.—The practical value of blood-counts, 234
Gordon Memorial College, Khartoum, 78
Gould and J. C. Warren, A. P. [Eds.]—The international text-book of surgery (R.), 61, 266.
Gould, Dr. G. M.—Biographic clinics (R.), 358.
Grant, Autobiography of Alexander (R.), 162.
Grant, Dr. H. H.—A text-book of surgical principles and surgical diseases of the face, mouth, and jaws for dental students (R.), 266.
Groves, Dr. E. W. H.—On the diagnosis of malignant disease of the body of the uterus, 128.
Grünwald, Dr. L.—Atlas and epitome of diseases of the mouth, pharynx, and nose (R.), 271.
Guy's hospital reports (R.), 68.
Gynæcology, Points of practical interest in—Dr. H. Macnaughton-Jones (R.), 62.
Gynæcology, Report on—Dr. W. C. Swayne, 153.

Hæmaturia caused by scurvy—Dr. R. S. Smith, 320.
Haines, Drs. F. Peterson and W. S. [Eds.]—A text-book of legal medicine and toxicology (R.), 267.

Hall, Dr. F. de H.—The medical examination for life assurance (R.), 363.
Harburn, W. Armstrong and J. E.—Buxton: its waters, &c. (R.), 368.
Harrison and W. K. Wills, Drs. A. J.—Remarks on the "light treatment" of lupus and rodent ulcer, etc., 23, 303.
Harrison, R.—The ambulance in civil life on land and sea (R.), 161.
Harsant, W. H.—Report on otology, 356.
Heath's practical anatomy (R.), 65.
Helferich, Prof.—Atlas and epitome of fractures and dislocations (R.), 368.
Hemiplegias, Common, 143.
Henderson, Dr. E.—The nurse in hot climates (R.), 274.
Hernias, Abdominal—Dr. G. Sultan (R.), 368.
Herschell, Dr. G.—Manual of intragastric technique (R.), 358.
Hewer, Mrs. J. L.—Our baby (R.), 271.
High-frequency currents, Medical use of, 256.
Hood, Dr. W. P.—The treatment of injuries by friction and movement (R.), 67.
Hygiene and public health.—Drs. L. Parkes and H. Kenwood (R.), 70.

Index-catalogue of the library of the Surgeon-General's Office, United States Army (R.), 274, 365.
Infantile scurvy—Dr. C. Elliott, 323; Dr. B. Rogers, 318.
Injuries, The treatment of—Dr. W. P. Hood (R.), 67.
International Congress of Medicine, The fourteenth, 165.
Intragastric techniqne—Dr. G. Herschell (R.), 358.

Joint disease, Missing links in—Dr. P. King, 137.
Jones, Dr. E. J. T.—Four cases of renal calculi, 245.
Jones, H. Macnaughton—*See* Macnaughton-Jones, H.
Journal of tuberculosis, The (R.), 274.

Kayser, R.—Anleitungen zur diagnose und therapie der kehlkopf-, nasen-, und ohrenkrankheiten (R.), 278.
Kehlkopf-, nasen-, und ohrenkrankheiten, Diagnose und therapie der —R. Kayser (R.), 278.
Kenwood, Dr. H. R.—Public health laboratory work (R.), 366.
Kenwood, Drs. L. Parkes and H.—Hygiene and public health (R.), 70.
King, Dr. P.—Missing links in joint disease, 137.
King's College hospital reports (R.), 364.
Kirkby, W.—The evolution of artificial mineral waters (R.), 61.
Kyle, Dr. H. G.—Perforated typhoid ulcer, 30.

Laboratory reports of the Royal College of Physicians of Edinburgh (R.), 365.
Laryngology, Report on—Dr. P. W. Williams, 354.
Larynx for malignant disease, Excision of the, 48.
Latham, Dr. A.—The diagnosis and modern treatment of pulmonary consumption (R.), 66.
Library of the Bristol Medico-Chirurgical Society, 81, 176, 284, 377.
Life assurance, Medical examination for—Dr. F. de H. Hall (R.), 363.
Light treatment, Remarks on the—Drs. A. J. Harrison and W. K. Wills, 23, 303; Dr. W. K. Wills, 119.
Local medical notes, 95, 192, 287, 383.
London, Transactions of the Medical Society of (R.), 68.
London, Transactions of the Obstetrical Society of (R.), 63.
Lung, Abscess of the, 44, 46.
Lupus, Light treatment of—Drs. A. J. Harrison and W. K. Wills, 23, 303; Dr. W. K. Wills, 119.

Mackenzie, Dr. J.—The study of the pulse (R.), 67.
Macnaughton-Jones, Dr. H.—Points of practical interest in gynæcology (R.), 62; [*et al.*]—Practitioner's handbook of diseases of the ear and naso-pharynx (R.), 59.
Malignant disease, Excision of the larynx for, 48.
Malignant disease of the body of the uterus—Dr E. W. H. Groves, 128.
Mann, Dr. J. D.—Forensic medicine and toxicology (R.), 64.
Manson, Dr. P.—Tropical diseases (R.), 275.
Marsh, H.—Clinical essays and lectures (R.), 57.
Massage—Dr. E. Ekgren (R.), 69.
Medical annual, The (R.), 274.
Medical Defence Union, 281.

Medicine, A manual of—Dr. W. H. Allchin [Ed.] (R.), 60; Dr. T. K. Monro (R.), 262.
Medicine, Reports on—Dr. J. M. Clarke, 345; Dr. T. Fisher, 44; Dr. G. Parker, 143, 248.
Medicine, The principles and practice of—Dr. W. Osler (R.), 66.
Medico-chirurgical transactions (R.), 272, 363.
Menstruation and its disorders—Dr. A. E. Giles (R.), 62.
Middle-ear disease, Suppurative, 356.
Mineral waters, Artificial—W. Kirkby (R.), 61.
Mobility—Prof. J. Chiene, 97.
Mole, H. F.—Two cases of ruptured tubal pregnancy, 39.
Monro, Dr. T. K.—A manual of medicine (R.), 262.
Moor, C. G.—Suggested standards of purity for foods and drugs (R.), 59.
Morris M.—Diseases of the skin (R.), 365.
Morton, C. A.—Report on surgery, 148.
Mosquitoes in Sierra Leone, The campaign against — Dr. M. L. Taylor (R.), 70.
Moullin, Dr. C. M.—The surgical treatment of ulcer of the stomach (R.), 277.
Mouth, Diseases of the—Dr. L. Grünwald (R.), 271.
Moynihan, A. W. M. Robson and B. G. A.—Diseases of the pancreas and their surgical treatment (R.), 55.
Muir and J. Ritchie, Drs. R.—Manual of bacteriology (R.), 70.
Murray, Dr. W.—Rough notes on remedies (R.), 69.
Muscular atrophy and sclerodermia —Dr. J. A. Nixon, 328.

Neff, Dr. M. L.—A brief manual of prescription writing (R.), 160.
New York, Transactions of the Medical Society of the State of (R.), 364.
Nixon, Dr. J. A.—Muscular atrophy and sclerodermia, 328.
Noorden, Prof. C. von—Diseases of metabolism and nutrition (R.), 159, 264.
Nose, A granuloma of the—Dr. C. P. Crouch, 231.
Nurse in hot climates, The—Dr. E. Henderson (R.), 274.
Nutrition, Diseases of metabolism and—Prof. C. von Noorden (R.), 159, 264.

Obituary, 94, 187, 193, 285.
Obstetrical Society of London, Transactions of the (R.), 63.
Obstetrics—J. W. Williams (R.), 359.
Obstetrics, Report on—Dr. W. C. Swayne, 153.
Ormerod, Obituary notice of Henry, 189.
Osler, Dr. W.—The principles and practice of medicine (R.), 66.
Otological Society of the United Kingdom, Transactions of the (R.), 161.
Otology, Report on—W. H. Harsant, 356.
Ovariotomy, Dangers of delay in—T. Carwardine, 309.

Pancreas, Surgical diseases of the 349; A. W. M. Robson and B. G. A. Moynihan (R.), 55.
Pancreatic secretions, 249.
Parker, Dr. G.—Reports on medicine, 143, 248.
Parkes and H. Kenwood, Drs. L.—Hygiene and public health (R.), 70.
Paterson, Dr. D. R.—The influence of the auricles on the percussion of the heart, 110.
Pawlow, Prof. J. P.—The work of the digestive glands (R.), 158.
Pediatrics—Dr. T. M. Rotch (R.), 64.
Peritonitis, Pneumococcal, 148.
Peterson and W. S. Haines, Drs. F. [Eds.] — A text-book of legal medicine and toxicology (R.), 267
Pharmacology and therapeutics, A text-book of—Dr. W. H. White (R.), 58.
Pharmacy, Practical—Dr. J. Calvert (R.), 366.
Philadelphia, Transactions of the College of Physicians of (R.), 276.
Physiology, Practical—W. Stirling (R.), 163.
Pleurisy, Initial signs of—Dr. F. H. Edgeworth, 36.
Pregnancy, Ruptured tubal—H. F. Mole, 39.
Prescription writing — Dr. M. L. Neff (R.), 160.
Prostatectomie périnéale pour hypertrophie—R. Proust (R.), 368.
Proust, R.—Manuel de la prostatectomie périnéale pour hypertrophie (R.), 368.
Public health laboratory work—Dr. H. R. Kenwood (R.), 366.
Pulse, The study of the—Dr. J. Mackenzie (R.), 67.

Remedies, Rough notes on—Dr. W. Murray (R.), 69.
Renal calculi, Four cases of—Dr. E. J. T. Jones, 245.
Respiration, Diseases of the organs of—Dr. S. West (R.), 54.
Respiratory tract as a source of systemic infection, The upper, 354.
Ritchie, Drs. R. Muir and J.—Manual of bacteriology (R.), 70.
Robson and B. G. A. Moynihan, A. W. M.—Diseases of the pancreas and their surgical treatment (R.), 55.
Roentgen-ray photographs in pathological conditions of the pancreas, 353.
Rogers, Dr. B. M. H.—A case of infantile scurvy, 318.
Rotch, Dr. T. M.—Pediatrics (R.), 64.
Roxburgh, Dr. R.—Therapeutics of a sea voyage, 1; Round the world, 202.
Rudge, Obituary notice of Henry Thomas, 190.

St. Bartholomew's hospital reports (R.), 364.
Sajous, Dr. C. E. de M.—The internal secretions and the principles of medicine (R.), 268.
Sanatorium, The King's, 372.
Sclerodermia, Muscular atrophy and —Dr. J. A. Nixon, 328.
Scudder, Dr. C. L.—The treatment of fractures (R.), 276.
Scurvy as a cause of hæmaturia—Dr. R. S. Smith, 320.
Scurvy, Infantile—Dr. C. Elliott, 323; Dr. B. M. H. Rogers, 318.
Sea voyage, Therapeutics of a—Dr. R. Roxburgh, 1, 75.
Secretions and the principles of medicine, The internal—Dr. C. E. de M. Sajous (R.), 268.
Sewers, Ventilation of, 73.
Shekleton, Obituary notice of Joseph Furlonge, 190.
Sick, Preparations for the, 79, 173, 281, 373.
Skin, Diseases of the—M. Morris (R.), 365.
Smith, Dr. R. S.—Note on scurvy as a cause of hæmaturia in the infant and in old age, 320.
Smith, G. [Ed.]—Physician and friend (R.), 162.
Smith, G. M.—A case of perforated duodenal ulcer, 218.
Smithsonian report (R.), 271.
Society, Bristol Medico-Chirurgical, 83, 179, 379.
Stackpoole, Florence—Advice to women (R.), 63.
Statton, Resignation of Mr. L., 78.
"Status lymphaticus," The, 345.
Stirling, W.—Outlines of practical physiology (R.), 163.
Stokes, Sir W.—Selected papers on operative and clinical surgery (R.), 56.
Stomach, Cancer and other tumours of the—Drs. S. and W. S. Fenwick (R.), 263.
Stomach, Ulcer of the—Dr. C. M. Moullin (R.), 277.
Sultan, Dr. G.—Atlas and epitome of abdominal hernias (R.), 368.
Surgery, International text-book of—A. P. Gould and J. C. Warren [Eds.] (R.), 61, 266.
Surgery, Operative—H. W. Allingham (R.), 265.
Surgery, Operative and clinical—Sir W. Stokes (R.), 56.
Surgery, Reports on.—T. Carwardine, 252; J. L. Firth, 48; C. A. Morton, 148; Dr. J. Swain, 349.
Surprises in my professional career, Some—J. P. Bush, 289.
Sutton, J. B.—Tumours, innocent and malignant (R.), 277.
Swain, Dr. J.—A contribution to the conservative surgery of the uterine appendages, 211; Report on surgery, 349.
Swayne, Dr. W. C.—Report on obstetrics and gynæcology, 153.
Swayne, Obituary notice of Joseph Griffiths, 193.
Symes, Dr. J. O.—Trypanosomiasis, 325.

Taylor, J.—Report on electro-therapeutics, 256.
Taylor, Dr. M. L.—Second progress report on the campaign against mosquitoes in Sierra Leone (R.), 70; Report of the sanitary conditions of Cape Coast Town (R.), 70.
Temporal bone, Operations on the, 356.
Tendon grafting for paralytic talipes 152.
Thrombosis, 147.
Tropical diseases.—Dr. P. Manson (R.), 275.
Trypanosomiasis—Dr. J. O. Symes, 325.
Tuberculosis—Dr. N. Bridge (R.), 360.

Tuberculosis, Bovine, 250, 281.
Tuberculosis statistics—Dr. T. A. Chapman (R.), 364.
Tumours—J. B. Sutton (R.), 277.
Tumours of the brain, Surgical treatment of, 348.
Typhoid fever, Etiology of—Dr. W. H. Corfield (R.), 366.
Typhoid ulcer, Perforated—Dr. H. G. Kyle, 30.

Underwood, A. S.—Aids to dental anatomy and physiology (R.), 164.
University College, Bristol, Opening address, 369.
University College Colston Society, 71.
Uterine appendages, The conservative surgery of the—Dr. J. Swain, 211.
Uterus, Malignant disease of the body of the—Dr. E. W. H. Groves, 128.

Vascular changes, 146.
Vermiform appendix, Primary carcinoma of the, 252.
Volkmann's contraction, 151.
Vomiting, Recurrent, 47.

Warren, A. P. Gould and J. C. [Eds.] —The international text-book of surgery (R.), 61, 266.
West, Dr. S.—Diseases of the organs of respiration (R.), 54.
Where shall I send my patient? (R.), 367.
White, Dr. W. H.—Text-book of pharmacology and therapeutics (R.), 58.
Williams, Dr. P. W.—On the probable rhythmical contraction of the bronchial muscular coat as a factor in pulmonary disease, 6; Report on laryngology, 354.
Williams, J. W.—Obstetrics (R.), 359.
Wills, Dr. W. K.—Some remarks upon the Finsen light treatment of lupus, 119.
Wills, Drs. A. J. Harrison and W. K. —Remarks on the "light treatment" of lupus and rodent ulcer, etc, 23, 303.
Windle, Dr. B. C. A.—A handbook of surface anatomy and landmarks (R.), 163.
Winsley sanatorium for consumptives, 75, 371; Laying the foundation stone, 170.
Women, Advice to—Florence Stackpoole (R.), 63.

Year-book of medicine and surgery, Saunders's (R.), 278, 365.
Year-book of scientific and learned societies (R.), 162.

J. W. Arrowsmith, Printer, Quay Street, Bristol.

### Bristol Medico-Chirurgical Society.

### PAST PRESIDENTS.

1874—75. FREDERICK BRITTAN, M.D., B.A. Dub.

1875—76. ROBERT WILLIAM COE.

1876—77. SAMUEL MARTYN, M.D. Ed.

1877—78. AUGUSTIN PRICHARD, M.D. Berlin.

1878—79. HENRY EDWARD FRIPP, M.D. Würzburg.

1879—80. WILLIAM MICHELL CLARKE.

1880—81. JOSEPH GRIFFITHS SWAYNE, M.D. Lond.

1881—82. EDWARD LONG FOX, M.D. Oxon.

1882—83. JAMES GEORGE DAVEY, M.D. St. And.

1883—84. WILLIAM JOHNSTONE FYFFE, M.D., B.A. Dub.

1884—85. GEORGE FORSTER BURDER, M.D. Aberd.

1885—86. WILLIAM HENRY SPENCER, M.D., M.A. Cantab.

1886—87. FRANCIS POOLE LANSDOWN.

1887—88. LEMUEL MATTHEWS GRIFFITHS.

1888—89. ROBERT SHINGLETON SMITH, M.D., B.Sc. Lond.

1889—90. NELSON CONGREVE DOBSON.

1890—91. SAMUEL HENRY SWAYNE.

1891—92. FRANCIS RICHARDSON CROSS, M.B. Lond.

1892—93. EDWARD MARKHAM SKERRITT, M.D., B.S., B.A. Lond.

1893—94. JAMES GREIG SMITH, M.B., C.M., M.A. Aberd., F.R.S.E.

1894—95. ALFRED JAMES HARRISON, M.B. Lond.

1895—96. ARTHUR WILLIAM PRICHARD.

1896—97. ALFRED EDWARD AUST LAWRENCE, M.D., C.M. Aberd.

1897—98. JOHN EDWARD SHAW, M.B., C.M. Ed.

1898—99. ROBERT ROXBURGH, M.B. Ed.

1899—00. WILLIAM HENRY HARSANT.

1900—01. DAVID SAMUEL DAVIES, M.D. Lond.

1901—02. BARCLAY JOSIAH BARON, M.B., C.M. Ed.

### 1903.

### LIST OF SUBSCRIBERS

TO THE

### Bristol Medico-Chirurgical Journal.

*Those marked * are Members of the Society.*

| | |
|---|---|
| ACKLAND, C. H. ... ... ... ... ... ... ... | Moorland Court, Poole Road, Bournemouth. |
| ACKLAND, J. MCKNO ... ... ... ... ... ... | 24 Southernhay, Exeter. |
| *ACKLAND, W. R. ... ... ... ... ... ... ... ... | 5 Rodney Place, Clifton. |
| ADYE, W. J. A. ... ... ... ... ... ... ... ... | Church House, Bradford, Wilts. |
| ALDOUS, GEORGE F.... ... ... ... ... ... ... | Charlton House, Mannamead, Plymouth. |
| ALDRIDGE, CHARLES, M.D., C.M. Aberd.... ... | Plympton. |
| *ALEXANDER, D.A., M.B., Ch.B. Vict.... ... ... | 30 Berkeley Square, Clifton. |
| ALEXANDER, JAMES, M.D., M.Ch., B.A. Dub.... | Bishop's Place, Paignton. |
| AMBROSE, J., M.B., C.M. Aberd. ... ... ... ... | 138 Fishponds Road, Eastville, Bristol. |
| AVELINE, H. T. S. ... ... ... ... ... ... ... | Somerset Asylum, Taunton. |

| | |
|---|---|
| *BALLANCE, H. STANLEY, M.D. Lond. ... ... | 4 Royal Terrace, Weston-super-Mare. |
| *BARCLAY, W. M. ... ... ... ... ... ... ... ... | 21 Oakfield Road, Clifton. |
| BARETTI, T. G. L'ENARDI ... ... ... ... ... ... | 49 Royal York Crescent, Clifton. |
| *BARKER, G. H., M.B., B.Sc. Lond. ... ... ... | 124 Redland Road, Bristol |
| *BARON, BARCLAY J., M.B., C.M. Ed.... ... ... | 16 Whiteladies Road, Clifton. |
| *BASSET, WALTER ... ... ... ... ... ... ... ... | 24 Stow Hill, Newport, Mon. |
| BATTEN, RAYNER W., M.D. Lond. ... ... ... | 1 Brunswick Square, Gloucester. |
| BATTEN, W. SMITH... ... ... ... ... ... ... | West Hill, Calne. |
| BAYER CO., LTD. ... ... ... ... ... ... ... | 20 Booth Street, Mosley Street, Manchester. |
| *BEDDOE, JOHN, M.D.Ed., B.A. Lond., F.R.S. | The Chantry, Bradford-on-Avon. |
| BENHAM, HARRY A., M.D., C.M. Aberd. ... ... | Asylum, Fishponds, Gloucestershire. |

### LIST OF SUBSCRIBERS.

| | |
|---|---|
| Bennett, W. S. ... ... ... ... ... ... ... | Escot, Penzance. |
| Bernard, C. ... ... ... ... ... ... ... ... | 2 Spencer Terrace, Fishponds. |
| *Berry, F. C., M.D., B.Ch., B.A. Dub. ... ... | Burnham, Somersetshire. |
| Berry, James, M.B., B.S. Lond. ... ... ... | 21 Wimpole Street, London, W. |
| Biamonti, Sebastiano ... ... ... ... ... ... | 27 Via Balbi, Genoa, Italy. |
| *Blacker, A. E.... ... ... ... ... ... ... ... | 20 Victoria Square, Clifton. |
| *Blagg, Arthur F., M.D. Durh.... ... ... ... | 28 Caledonia Place, Clifton. |
| Boyce, James G. ... ... ... ... ... ... ... | The Chipping, Wotton-under-Edge. |
| Boyd, Geo. S. J. ... ... ... ... ... ... ... | 10 Adelaide Terrace, Portishead. |
| Braine-Hartnell, J. C. R.... ... ... ... ... | Cotswold Sanatorium, Cranham, near Stroud. |
| *Brasher, Charles W. J. ... ... ... ... ... | 128 Cotham Brow, Bristol. |
| Briggs, H. Fielden, D.D.S. Mich.... ... ... | 20 Belgrave Road, Clifton. |
| *Bristowe, H. C., M.D. Lond. ... ... ... ... | Wrington, Somerset. |
| Brown, G. Arthur ... ... ... ... ... ... | Tredegar. |
| Brown, William, M.D., C.M. Glasg. ... ... | Park View, Fishponds, Gloucestershire. |
| Browne, J. Walton, M.D., B.A. Royal Univ. I. | 10 College Square North, Belfast. |
| *Bullen, F. St. John ... ... ... ... ... ... | 12 Pembroke Road, Clifton. |
| *Bunbury, E. G. ... .. ... ... ... ... ... | 13 Dowry Square, Hotwells. |
| Burroughs, E. F. H. ... ... ... ... ... ... | 3 Elgin Park, Bristol. |
| *Bush, J. Paul, C.M.G. ... ... ... ... ... ... | Vyvyan House, Clifton Park, Clifton. |

| | |
|---|---|
| *Carr, Alexander ... ... ... ... ... ... ... | 150 Wells Road, Bristol. |
| *Carter, T. M. ... ... ... ... ... ... ... ... | 28 Victoria Square, Clifton. |
| *Carwardine, Thomas, M.B., M.S. Lond. ... | 16 Victoria Square, Clifton. |
| *Cave, Edward J., M.D. Lond. ... ... ... ... | 20 Circus, Bath. |
| Chute, Henry M. ... ... ... ... ... ... ... | King William's Town, S. Africa. |
| *Clarke, John Michell, M.D., M.A. Cantab. | 28 Pembroke Road, Clifton. |
| Cochran, Alexander, M.B., C.M. Glasg. ... | Bath Road, New Brislington, Bristol. |
| *Collinson, T. A. ... ... ... ... ... ... ... | 170 Cheltenham Road, Bristol. |
| *Cook, Henri, M.D., C.M. Aberd. ... ... ... | 1 Dean Lane, Southville, Bristol. |
| Cornall, Miss ... ... ... ... ... ... ... ... | 20 Arley Hill, Bristol. |
| *Corrigan, W. J. ... ... ... ... ... ... ... | Cloughmore, Splott Avenue, Cardiff. |
| Cossham, W. R., M.D., C.M. Aberd.... ... ... | Cirencester. |
| Cotton, William, M.B., C.M., M.A. Ed. ... | 231 Gloucester Road, Bristol. |
| Coulter, R. J., M.B. Dub. ... ... ... ... ... | Warwick House, Stow Hill, Newport, Mon. |
| Cousins, J. Ward, M.D. Lond. ... ... ... ... | Kent Road, Southsea. |
| Cowan, F. S. ... ... ... ... ... ... ... ... | 27 Queen Square, Bath. |
| Craddock, Samuel... ... ... ... ... ... ... | South Lyncombe, Bath. |
| *Cridland, A. B. ... ... ... ... ... ... ... | 9 Westbury Road, Bristol. |
| Crisp, J. Ellis ... ... ... ... ... ... ... ... | Alexander House, Corsham. |
| *Cross, F. Richardson, M.B. Lond.... ... ... | Worcester House, Clifton. |
| *Crossman, Edward, M.D. Dur.... ... ... ... | Hambrook, Gloucestershire. |
| *Crossman, F. W. ... ... ... ... ... ... | Hambrook, Gloucestershire. |
| Crouch, C. Percival, M.B. Lond. ... ... ... | 1 Royal Terrace Weston-super-Mare. |
| Culross, James, M.B., C.M., M.A. Glasg. ... | 66 Queen Street, Newton Abbot. |

| | |
|---|---|
| *Dacre, John ... ... ... ... ... ... ... ... | 14 Eaton Crescent, Clifton. |
| Dalby, Augustus W. ... ... ... ... ... ... | Argyll House, Frome. |
| *Davies, D. S., M.D. Lond., D.P.H. Cantab. ... | 60 Oakfield Road, Clifton. |
| Davis, Theodore, M.D. Lond. ... ... ... ... | Beachcroft, Clevedon. |

### LIST OF SUBSCRIBERS.

| | |
|---|---|
| DAVY, HENRY, M.D. Lond ... ... ... ... ... ... | Southernhay House, Exeter. |
| DENING, EDWIN ... ... ... ... ... ... ... ... | Manor House, Stow-on-the-Wold. |
| DICKIE, JAMES STEEL, M.D., C.M. Aberd. ... | Boscombe Park, Bournemouth. |
| *DOBSON, NELSON C. ... ... ... ... ... ... | Avonmore, Sneyd Park. |
| DOWN, A. R. ... ... ... ... ... ... ... ... ... | Bampton, Devon. |
| DUNBAR, ELIZA L. WALKER, M.D. Zürich ... | 9 Oakfield Road, Clifton. |

| | |
|---|---|
| EADON, W. F. BAILEY ... ... ... ... ... ... | Hambrook, Gloucestershire. |
| *EAGER, REGINALD, M.D. Lond. ... ... ... ... | Northwoods, Winterbourne. |
| *EAGER, W. R. ... ... ... ... ... ... ... ... ... | Northwoods, Winterbourne. |
| EBERLE, EMILY, M.A., M.B., B.Ch. Dub. ... | 17 Oakfield Road, Clifton. |
| *EDGEWORTH, F. H., M.B., B.C., B.A. Cantab., B.Sc. Lond. ... ... ... ... ... ... ... ... | 35 Pembroke Road, Clifton. |
| ELDER, WILLIAM ... ... ... ... ... ... ... ... | 4 Trelawney Road, Bristol. |
| *ELLIOTT, CHRISTOPHER, M.D., B.A. Dub. ... | 88 Pembroke Road, Clifton. |
| ELLIOTT, F. PERCY, M.B., C.M. Aberd. ... ... | 113 Grove Road, Walthamstow, London, N.E. |
| ELLIS, W. McD., M.D. Brux. ... ... ... ... | Cedar Lodge, Bath. |
| EVANS, T. G. C. ... ... ... ... ... ... ... | Budleigh Salterton, Devon. |

| | |
|---|---|
| FAIRBANKS, W., M.D., C.M. Ed. ... ... ... ... ... | Wells, Somersetshire. |
| *FAWCETT, E., M.B., C.M. Ed. ... ... ... ... ... | 141 Redland Road, Bristol. |
| *FENDICK, R. GEORGE ... ... ... ... ... ... ... | St. Paul's Road, Clifton. |
| *FERGUSON, R. S , B.A. Durh., M.B. C.M. Edin. | Elm Grove, Calne, Wilts. |
| *FIRTH, J. L., M.D., M.S. Lond. ... ... ... ... | 20 Richmond Park Road, Clifton. |
| *FISHER, THEODORE, M.D. Lond.... ... ... ... | Harley Lodge, Clifton. |
| *FLEMMING, A. L. ... ... ... ... ... ... ... | Richmond Park Road, Clifton. |
| *FLEMMING, C. E. S. ... ... ... ... ... ... | Manvers House, Bradford-on-Avon. |
| *FLETCHER, J., M.B., C.M. Aberd. ... ... ... | Ham Green, Somerset. |
| FLOOD, P. ... ... ... ... ... ... ... ... ... ... ... | 318 Stapleton Road, Bristol. |
| FORD, JOSEPH ... ... ... ... ... ... ... ... ... | Wedmore. |
| *FORTESCUE-BRICKDALE, J. M., M.A., M.D., B.Ch. Oxon.... ... ... ... ... ... ... ... ... | 52 Pembroke Road, Clifton. |
| FORTY, D. H. ... ... ... ... ... ... ... ... ... | Wotton-under-Edge. |
| FOSS, E. V. ... ... ... ... ... ... ... ... ... | Babbacombe House, Ashton Road, Bedminster, Bristol. |
| FOWLER, O. H. ... ... ... ... ... ... ... ... ... | Cirencester. |
| FOX, F. F. ... ... ... ... ... ... ... ... ... ... | Yate House, Chipping Sodbury. |
| FOX, T. COLCOTT, M.B. Lond., B.A. Cantab.... | 14 Harley Street, London, W. |
| *FRASER, FORBES ... ... ... ... ... ... ... | 2 The Circus, Bath. |
| FRAZER-TOOVEY, T. E. ... ... ... ... ... ... | The Hospital, Teignmouth. |
| FREEMAN, J., M.D. Brux. ... ... ... ... ... | Glencairn Villa, Queen's Road, Clifton. |

| | |
|---|---|
| GAMBLE, C. H. ... ... ... ... ... ... ... ... ... | Barnstaple. |
| GARLAND, E. B., M.B., C.M. Edin. ... ... ... | 92 Hampton Road, Redland. |
| GENGE, T. T. ... ... ... ... ... ... ... ... ... | 21 Whiteladies Road, Bristol. |
| GENT, W. C. ... ... ... ... ... ... ... ... ... | 170 Wells Road, Bristol. |
| *GERRISH, D. S. ... ... ... ... ... ... ... ... | 322 Church Road, St. George, Bristol. |
| *GIBBS, ALFRED N. GODBY ... ... ... ... ... | 52 Whiteladies Road, Clifton. |
| GIDLEY, G. G. ... ... ... ... ... ... ... ... ... | Cullompton, Devon. |
| GOSS, T. BIDDULPH... ... ... ... ... ... ... | 1 Circus, Bath. |

### LIST OF SUBSCRIBERS.

Grace, Alfred ... ... ... ... ... ... ... ... Chipping Sodbury.

Grace, Edward Mills... ... ... ... ... ... Thornbury, Gloucestershire

Gratte, C. Brooke ... ... ... ... ... ... 183 Commercial Road, Newport, Mon.

*Greer, W. J. ... ... ... ... ... ... ... ... 2 Chepstow Road, Newport, Mon.

Grey, T. C. ... ... ... ... ... ... ... ... Camborne, Cornwall.

*Griffiths, J. S. ... ... ... ... ... ... ... 25 Redland Park, Bristol.

*Griffiths, L. M. ... ... ... ... ... ... ... 11 Pembroke Road, Clifton.

*Groves, E. W. H., M.B., B.Sc. Lond. ... ... Kingswood, Bristol.

Hadwen, W. R., M.D. St. And.... ... ... ... 35 Brunswick Square, Gloucester.

*Hailes, Clements D. G., M.D., C.M. Ed. ... 27 Alma Road, Clifton.

*Haines, A. H. ... ... ... ... ... ... ... ... 8 Cotham Road, Bristol.

*Hall, E. G., M.B. Lond. ... ... ... ... ... 53 Hampton Road, Redland, Bristol.

Hamilton, D. L. ... ... ... ... ... ... ... Timsbury, near Bath.

Handfield-Jones, Montagu, M.D. Lond. ... 35 Cavendish Square, London, W.

*Harris, H. Elwin, B.A., M.B. Cantab. ... ... 13 Lansdown Place, Clifton.

*Harrison, A. J., M.B. Lond. ... ... ... ... Guthrie Road, Clifton.

Harsant, J. G., M.D., B.S. Lond. ... ... ... Exeter Road, Bournemouth.

*Harsant, W. H. ... ... ... ... ... ... ... Tower House, Clifton.

*Harvey, Alfred, M.B. Lond. ... ... ... ... Westbury-on-Trym.

Hay, M. B.... ... ... ... ... ... ... ... ... North Devon Infirmary, Barnstaple.

*Haydon, H. W., M.B. Durh. ... ... ... ... Beaufort House, Park Row, Bristol.

Hayman, Alfred G. ... ... ... ... ... ... Hapsford House, near Frome.

*Hayman, C. A., M.D. St. And. ... ... ... ... Kingston Villa, Richmond Hill, Clifton.

Heath, Miss ... ... ... ... ... ... ... ... Isolation Hospital, Taunton.

*Heaven, John C., D.P.H. Lond. ... ... ... 17 Whiteladies Road, Bristol.

*Herapath, C. K. C. ... ... ... ... ... ... Cotham Park, Bristol.

*Hetling, H. E. ... ... ... ... ... ... ... 30 Elliston Road, Bristol.

Hill, Hedley ... ... ... ... ... ... ... ... ... 1 Redcliff Hill, Bristol.

*Hill, Thomas, M.D. St. And. ... ... ... ... 138 Cromwell Road, Bristol.

*Hill, Walter J. ... ... ... ... ... ... ... Edgcumbe Villa, Clevedon.

Hoffman, A. W. W. ... ... ... ... ... ... 30 The Parade, Leamington.

Hospital, Bristol General (*Sec.*, W. Thwaites).

Hospital, Taunton and Somerset (*House-Surgeon*, W. Drake).

Howells, William, M.B., C.M. Glasg. ... ... Watton House, Brecon.

Huggard, W. R., M.D., M.Ch., M.A. Roy. Univ. I.... ... ... ... ... ... ... ... ... Davos-Platz, Switzerland.

Hunt, A. D. ... ... ... ... ... ... ... ... Millbrook House, Chagford.

Imlay, G. A., M.B., C.M. Aberd. ... ... ... 1 Cotham Park, Bristol.

Ingle, C. Durban ... ... ... ... ... ... ... Somerton, Somerset.

Ives, William R. Y. ... ... ... ... ... ... 14 Portswood Park, Bevois Hill, Southampton.

Jackson, Mark, M.D. Roy. Univ. I. ... ... ... Bear Street, Barnstaple.
Jacobson, W. H. A., M.B., M.C., M.A. Oxon. 66 Great Cumberland Place, London, W.
Jamison, A., M.B., B.Ch., M.A. Roy. Univ. I. ... 66 Woodstock Road, Belfast.
Jones, Evan ... ... ... ... ... ... ... ... Ty-mawr, Aberdare.
*Jones, E. J. Trevor, M.D. Brux. ... ... ... Aberdare, Glamorganshire.
Jones, H. Macnaughton, M.D., M.Ch. Roy. U.I.... ... ... ... ... ... ... ... ... ... 131 Harley Street, London, W.
Jones, W. W., M.B., C.M. Ed. ... ... ... ... ...47 Wellington Street, Merthyr Tydvil.

| | |
|---|---|
| *KEMM, F. ST. J.... ... ... ... ... ... ... ... | Worle. |
| *KENDALL, H. W. ... ... ... ... ... ... ... | 1 Burlington Road, Redland. |
| KENNEDY, W. P., M.D., B.Ch., B.A. Dub. ... | High Street, Lydney. |
| KINNEIR, R. ... ... ... ... ... ... ... ... | The Tower House, Malmesbury. |
| *KYLE, H. G., M.B., B.Ch. Oxon. ... ... ... | 53 Pembroke Road, Clifton. |

| | |
|---|---|
| *LACE, F. ... ... ... ... ... ... ... ... ... | 1 Paragon, Bath. |
| LANCASTER, E. LE CRONIER, B.A., M.B., B.Ch. Oxon. ... ... ... ... ... ... ... ... ... | Winchester House, Swansea. |
| *LANSDOWN, F. POOLE ... ... ... ... ... ... | 19 Whiteladies Road, Clifton. |
| *LANSDOWN, R. G. P., M.B., B.S. Dur. ... ... | 39 Oakfield Road, Clifton. |
| LAWRIE, J. MACPHERSON, M.D., C.M. Glasg.... | Greenhill, Weymouth. |
| LE SOUDIER, H. ... ... ... ... ... ... ... | 174 et 176 Boulevard St. Germain, Paris. |
| *LEES, E. LEONARD, M.D., C.M. Ed. ... ... ... | 1 Chesterfield Place, Clifton. |
| LEIGH, W. W. ... ... ... ... ... ... ... ... | Treharris, Glamorganshire. |
| *LEONARD, R. C., M.D. Ed. ... ... ... ... ... | 134 Coronation Road, Bristol. |
| *LIGERTWOOD, WALTER ... ... ... ... ... ... | Bath and Somerset County Asylum, Wells. |
| LISTER, LORD, P.R.S. ... ... ... ... ... ... | 12 Park Crescent, London, N.W. |
| LOWE, T. PAGAN ... ... ... ... ... ... ... | 16 Circus, Bath. |
| *LUCAS, J. J. S. ... ... ... ... ... ... ... ... | 186 Wells Road, Totterdown. |
| LUCY, REGINALD H. ... ... ... ... ... ... | 9 The Crescent, Plymouth. |

| | |
|---|---|
| *McCREA, P. W., M.D., B.Ch., B.A.O., B.A. Dub. ... ... ... ... ... ... ... ... ... | 19 Portland Square, Bristol. |
| MAC DONALD, P. W., M.D., C.M. Aberd.... ... | County Asylum, Dorchester. |
| *MC KEE, A. B., M.B., B.Ch. Dub. ... ... ... | Sussex Villa, Sussex Place, Bristol. |
| MACKINNON, CHARLES, M.B., C.M., M.A. Glasg. | Davaar, Cirencester. |
| MACLEAN, EWEN J., M.D., C.M. Ed.... ... ... | 12 Park Place, Cardiff. |
| MACLEAN, J. CAMPBELL, M.B., C.M. Ed.... ... | Swindon House, Swindon, Wiltshire. |
| MACREADY, J. ... ... ... ... ... ... ... ... | 42 Devonshire Street, Portland Place, London, W. |
| MC WATTERS, J. C. ... ... ... ... ... ... | Almondsbury. |
| MARSH, O. E. BULWER ... ... ... ... ... ... | Parkdale, Newport, Mon. |
| MARSHALL, ARTHUR L., M.B. B.A. Cantab ... | 206 Upper Clapton Road, London, N.E. |
| *MARTIN, A. F., M.B., Ch.B. Vic.... ... ... ... | North Staffordshire Infirmary, Stoke-upon-Trent. |
| *MARTIN, E. F., M.B. Ed. ... ... ... ... ... | Victoria House, Weston-super-Mare. |
| *MARTIN, R. C. ... ... ... ... ... ... ... ... | 3 Clifton Vale, Clifton. |
| *MARTYN, G. J. KING, M.D., B.C., B.A. Cantab. ... ... ... ... ... ... ... ... ... | 12 Gay Street, Bath. |
| MASON, S. B. ... ... ... ... ... ... ... ... | 12 Park Terrace, Pontypool. |
| MASON, WILLIAM ... ... ... ... ... ... ... | Sedgemoor Cottage, St. Austell. |
| MATTHEWS, CHARLES E., M.D. Dur. ... ... | 6 Hickman Road, Penarth. |
| MATTHEWS, H. E. ... ... ... ... ... ... ... | The Mall Pharmacy, Clifton. |
| *MELSOME, W. S., M.D. Cantab.... ... ... ... | 29 Circus, Bath. |
| MERCK, E. ... ... ... ... ... ... ... ... ... | 16 Jewry Street, London, E.C. |
| MEREDITH, JOHN, M.D. Ed. ... ... ... ... ... | Wellington, Somersetshire. |

| | |
|---|---|
| *Millard, W. J. Kelson, M.D. St. And ... ... | 7 Bayshill Villas, Cheltenham. |
| Mitchell, Trafford, M.D. ... ... ... ... | Gorseinon, R.S.O., Glamorganshire. |
| *Mole, Harold F. ... ... ... ... ... ... ... ... | 1 Tottenham Place, Clifton, Bristol. |
| Monckton, William ... ... ... ... ... ... ... | Portishead. |
| Moores, De la Hey ... ... ... ... .. ... | 6 West Mall, Clifton. |
| Morgan, Albert T., M.D. Brux. ... ... ... | 89 Chesterfield Road, St. Andrew's Park, Bristol. |
| *Morgan, T. Whitworth S. ... ... ... ... | Pill. |
| *Morton, C. A. ... ... ... ... ... ... ... ... ... | 14 Vyvyan Terrace, Clifton. |
| *Morton, W. B., M.D. Lond. ... ... ... ... | Brislington House, Brislington. |
| Munden, Charles ... ... ... ... ... ... ... ... | Ilminster. |
| *Myles, G T. ... ... ... ... ... ... ... ... ... | 147 Whiteladies Road, Bristol. |

| | |
|---|---|
| *Neild, Newman, M.B., B.Ch. Vict. ... ... ... | 9 Richmond Hill, Clifton. |
| *Nevill, W. N., M.D., B.Ch., B.A. Dub. ... ... | Leighton Villa, Southville, Bristol. |
| *Newman, Charles ... ... ... ... ... ... ... ... | 156 Cheltenham Road, Bristol. |
| *Newnham, W. H. C., M.B., M.A. Cantab. ... | Chandos Villa, Clifton. |
| Nicholson, T. D., M.D. Ed. ... ... ... ... | 2 Whiteladies Road, Clifton. |
| *Nixon, J. A., B.A., M.B., B.C. Cantab. ... ... | Bristol Royal Infirmary. |
| Norman, J. H. ... ... ... ... ... ... ... ... ... | Goodleigh, Winkleigh, Devonshire. |

| | |
|---|---|
| O'Brien, Cornelius ... ... ... ... ... ... ... | Devon House, Whitehall, Bristol. |
| Odell, William, M.D. Durh. ... ... ... ... | Ferndale, Torquay. |
| *Ogilvy, Alexander, M.D., B.Ch., B.A. Dub. | Montrose House, Queen's Road, Clifton. |
| Openshaw, E. H. ... ... ... ... ... ... ... ... | Cheddar. |
| *Ormerod, H. Lawrence, M.B., B.Ch., B.A.O., R.U.I. ... ... ... ... ... ... ... ... ... | Burnley, Westbury-on-Trym |

| | |
|---|---|
| Page, G. Shepley ... ... ... ... ... ... ... ... | 78 Old Market Street, Bristol. |
| *Parker, George, M.D., M.A. Cantab. ... ... | 14 Pembroke Road, Clifton. |
| Parry, J. H. ... ... ... ... ... ... ... ... ... | Ashley Road, Bristol. |
| Parsons, Francis J. C.... ... ... ... ... ... ... | Ivy House, Bridgwater. |
| *Parsons, H. F.... ... ... ... ... ... ... ... ... | The Heath, Stoke Bishop. |
| Paterson, Donald Rose, M.D., C.M. Ed. ... | 18 Windsor Place, Cardiff. |
| Peake, Arthur W.... ... ... ... ... ... ... ... | 34 Gloucester Road, Bishopston, Bristol. |
| Peake, G. Arthur ... ... ... ... ... ... ... ... | Rodney Place, Cheltenham. |
| *Pearse, E. M. ... ... ... ... ... ... ... ... ... | 51 Clarendon Road, Redland, Bristol. |
| Peck, Major, R.A.M.C. ... ... ... ... ... ... | United Service Club, Calcutta. |
| *Phillips, C. M., M.D. Brux. ... ... ... ... | Ravenslea, Kensington Hill, Brislington. |
| Phillips, Edward Picton ... ... ... ... ... | High Street, Haverfordwest. |
| *Pickering, C. F. ... ... ... ... ... ... ... ... | 13 Berkeley Square, Bristol. |
| Plaister, W. H. ... ... ... ... ... ... ... ... | High Road, Tottenham, N. |
| Pollard, Reginald, M.B. Dur. ... ... ... | 8 Higher Terrace, Torquay. |
| *Pountney, W. E., M.D., C.M. Ed. ... ... ... | 33a Whiteladies Road, Bristol. |
| Powell, J. J., M.D. Lond. ... ... ... ... ... | 2 Portland Square, Bristol. |

### LIST OF SUBSCRIBERS.

| | |
|---|---|
| *Powell, L. W. ... ... ... ... ... ... ... ... ... | Two Mile Hill Road, Kingswood, Bristol. |
| Powell, William, M.B. Lond. ... ... ... ... | Hill Garden, Torquay. |
| *Prichard, Arthur W. ... ... ... ... ... ... ... | 6 Rodney Place, Clifton. |
| *Prowse, Arthur B., M.D. Lond. ... ... ... | 5 Lansdown Place, Clifton. |
| Pruen, S. T., M.D. Dur. ... ... ... ... ... ... | Sherborne Lodge, Cheltenham. |
| Purdy, J. R., M.D. Aber. ... ... ... ... ... ... | Lower Hutt, New Zealand. |
| Randolph, Charles ... ... ... ... ... ... ... | St. Michael's, Milverton, Somersetshire. |
| *Rattray, J. M., M.D., C.M., M.A. Aberd. ... | Frome. |
| *Rayner, D. C. ... ... ... ... ... ... ... ... ... ... | 7 Victoria Square, Clifton, Bristol. |
| Redwood, T. Hall, M.D., C.M., M.A. Dur. ... | The Lawn, Rhymney, Monmouthshire. |
| Rendall, John ... ... ... ... ... ... ... ... | Forestside, Lymington. |
| Richards, D. T. ... ... ... ... ... ... ... ... ... | The Meadows, Risca, Mon. |
| Riddell, Andrew W. ... ... ... ... ... ... ... | New Brighton, Cheshire. |
| Roberts, Frederick T., M.D., B.Sc. Lond. | 102 Harley Street, London, W. |
| Rodgers, John W., M.B., C.M. Ed. ... ... | Redfield, Gloucestershire. |
| *Rogers, B. M. H., M.D., B.Ch., B.A. Oxon. ... | 1 Victoria Square, Clifton. |
| *Rose, F. H. ... ... ... ... ... ... ... ... ... ... | 13 Westfield Park, Clifton. |
| *Rossiter, George F., M.B. Lond. ... ... | Cairo Lodge, Weston-super-Mare. |
| *Roué, W. Barrett, M.D., M.S. Dur. ... ... | 2 Buckingham Place, Clifton. |
| Routh, R. Henry F. ... ... ... ... ... ... ... | Bridgwater. |
| *Roxburgh, Robert, M.B. Ed. ... ... ... | 1 Victoria Buildings, Weston-super-Mare. |
| *Rudge, C. King ... ... ... ... ... ... ... ... | 145 Whiteladies Road, Bristol. |
| *Rudge, H. T. ... ... ... ... ... ... ... ... ... | 5 Colston Parade, Bristol. |
| Ruffer, M. Armand, M.D., B.S., M.B. Oxon. | Alexandria, Egypt. |
| Rutherford, H. T., M.A., M.D. Camb. ... ... | Taunton. |
| *Scott, Edward, M.D. Durh. ... ... ... ... ... | 25 York Crescent Road, Clifton. |
| Scott, Richard J. H. ... ... ... ... ... ... ... | 28 Circus, Bath. |
| Searle, George C. ... ... ... ... ... ... ... | Brixham, Devonshire. |
| Searle, Richard Burford... ... ... ... .. ... | Bexley, Dartmouth. |
| *Semple, W. R. M., M.B., C.M. Glasg. ... ... | Beltrees, Yeovil, Somersetshire. |
| Seward, W. J., M.B. Lond. ... ... ... ... ... | Asylum, Colney Hatch, London, N. |
| *Shaw, J. E., M.B., C.M. Ed. ... ... ... ... | 23 Caledonia Place, Clifton. |
| Shaw, Jesse, M.D.... ... ... ... ... ... ... ... | Fort Beaufort, Cape Colony. |
| Shaw-Mackenzie, John A., M.D. Lond. ... | 31 Grosvenor Street, Grosvenor Square, W. |
| Sheen, A., M.D. St. And. ... ... ... ... ... ... | 23 Newport Road, Cardiff. |
| *Sheppard, E. J. .. ... ... ... .. ... ... | 8 Richmond Hill, Clifton. |
| *Shoolbred, W. A. ... ... ... ... ... ... ... ... | St. Ann's, Chepstow. |
| Simons, R. J. ... ... ... ... ... ... ... ... ... | Nolton Court, Bridgend. |
| Skelton, Henry, M.D. St. And. ... ... ... | Downend, Gloucestershire. |
| *Skerritt, E. Markham, M.D., B.S., B.A. Lond. | Richmond Hill, Clifton. |
| *Smith, G. Cockburn, M.D. Brux. ... ... ... | 1 Chantry Road, Clifton. |
| *Smith, G. Munro ... ... ... ... ... ... ... ... | 18 Apsley Road, Clifton. |
| Smith, James ... ... ... ... ... ... ... ... ... | Meridian Road, Redland. |
| Smith, Rev. Reginald, M.A. Cantab. ... ... | The Vicarage, Walpole St. Andrew, Wisbech, Norfolk. |
| *Smith, R. Shingleton, M.D., B.Sc. Lond. ... | Clifton Park, Clifton. |
| Smith, W., M.B. Lond.... ... ... ... ... ... ... | Brabourne, Ashford, Kent. |
| Smith, W. Alexander, M.B., M.A. Oxon. ... | Newport, Essex. |
| *Smith, W. A. L., M.A., M.B., B.C. Cantab. ... | 8 Chamberlain Street, Wells. |
| Smyth, H. J. ... ... ... ... ... ... ... ... ... | New Road, South Molton. |
| Smyth, J. C. ... ... ... ... ... ... ... ... ... | |

Society, Cardiff Medical (*Hon. Lib.*, Queen Street, Cardiff).
Society, Manchester Medical ... ... ... Medical Society's Library, The Owens College, Manchester.
Society, Plymouth Medical (*Hon. Lib.*, J. Elliott Square).
Solly, S. E. ... ... ... ... ... ... ... ... Colorado Springs, Colorado, U. S. A.
*Stack, E. H. E., B.A., M.B., B.C. Cantab. ... Royal Infirmary, Bristol.
Stamp, W. D. ... ... ... ... ... ... ... ... Ridgeway House, Plympton.
Staple, J. D. ... ... ... ... ... ... ... ... Clevedon Villa, Cheltenham Road, Bristol.
Statham, R. Whiteside ... ... ... ... ... Cheddar, Somersetshire.
*Steele, Charles, M.D. Dur. ... ... ... ... 17 Richmond Hill, Clifton.
*Steele, W. H., Lt.-Col., M.D. Dub. ... ... 18 Mortimer Road, Clifton.
*Stephenson, J. B., M.B., B.S. Dur. ... ... 19 Park Row, Bristol.
Stevens, W. H. ... ... ... ... ... ... ... Zetland Road, Bristol.
*Stewart, J., B.A., R.U.I. ... ... ... ... ... Dunmurry, Sneyd Park, Bristol.
Stock, P. G., R.A.M.C.... ... ... ... ... ... c/o P.M.O., South Africa.
*Stock, W. S. V., M.B., B.S. Lond. ... ... ... Bristol General Hospital.
Stoddart, F. Wallis ... ... ... ... ... ... Southey House, College Green, Bristol.
*Style, H., M.A., M.B., B.C. Cantab. ... ... Avonmouth.
*Swain, James, M.D., M.S. Lond. ... ... ... 4 Victoria Square, Clifton.
*Swayne, J. G., M.D. Lond. ... ... ... ... ... 74 Pembroke Road, Clifton.
*Swayne, W. Carless, M.D. Lond. ... ... ... St. Paul's Road, Clifton.
*Symes, J. O., M.D. Lond. ... ... ... ... ... 11 Richmond Hill, Clifton.
Symonds, H. P. ... ... ... ... ... ... ... 35 Beaumont Street, Oxford.
Symons, John ... ... ... ... ... ... ... ... Buriton House, Penzance.

Taylor, C. Bell, M.D. Edin. ... ... ... ... 9 Park Row, Nottingham.

*Taylor, James ... ... ... ... ... ... ... ... 36 Alma Road, Clifton.

*Temple, G. H., M.B., C.M. Ed. ... ... ... Weston-super-Mare.

Thomas, A. Garrod, M.D., C.M. Ed. ... ... Clytha Park, Newport, Mon.

Thomas, J. Lynn ... ... ... ... ... ... ... 21 Windsor Place, Cardiff.

Thomas, Raglan, M.D. Lond. ... ... ... ... 13 West Southernhay, Exeter.

Thomson, Eustace B., M.D., C.M. Glasg. ... Albany Place, Plymouth.

*Tivy, W. J.... ... ... ... ... ... ... ... ... 8 Lansdown Place, Clifton.

Tosswill, Louis H., M.B., B.A. Cantab.... ... 1 West Southernhay, Exeter.

Trevithick, Edgar, M.A., M.D. Cantab. ... 24 Promenade, Cheltenham.

Tyley, R. P., M.D. St. And.... ... ... ... ... Wedmore.

Vachell, Herbert R., M.D., C.M. Aberd. ... 18 Newport Road, Cardiff.
Vereker, R. H. ... ... ... ... ... ... ... Eastleigh, Curry Rivel, Somerset.

*Waldo, Henry, M.D., C.M. Aberd. ... ... ... 19 Pembroke Road, Clifton.

*Walker, Cyril H., M.B., B.A. Cantab. ... ... 16 Pembroke Road, Clifton.

Wallace, Rev. Canon, M.A. Oxon. ... ... ... 3 Gloucester Row, Clifton.

Wallace, J., M.B. ... ... ... ... ... ... ... 112 York Road, Bedminster.

*Wallace, John ... ... ... ... ... ... ... 1 Oriel Terrace, Weston-super-Mare.

Walsh, Leslie H., M.B., B.S. Durh. ... ... 21 Gay Street, Bath.

Ward, J. L. W.... ... ... ... ... ... ... ... Victoria Street, Merthyr Tydvil.

*Wathen, J. Hancocke ... ... ... ... ... ... 16 York Place, Clifton.

Watters, George T. B., M.D., C.M. Ed. ... Stonehouse, Gloucestershire.

Weatherly, Lionel A., M.D., C.M. Aberd. Bailbrook House, Bath.

| | |
|---|---|
| WEBB, J. B. | 10 Upper Barton Hill Road, Barton Hill, Bristol. |
| WEBBER, WILLIAM W. | Crewkerne. |
| *WHICHER, A. H. | 37 Gordon Road, Clifton. |
| WHITE, C. R. | Nixon Villa, Merthyr Vale, Glamorgan. |
| WHITE, J. W. | Orchard House, Nailsea. |
| WICKHAM, W. | Hill House, Tetbury. |
| WIGAN, C. A., M.D. Dur. | Portishead. |
| WIGMORE, J., M.D. Roy. Univ. I. | 20 Green Park, Bath. |
| WILCOX, HENRY, M.B. Aberd. | Fleet, Hampshire. |
| WILKIN, GRIFFITH C. | Blakesley, Nr. Towcester, North Hants. |
| WILLCOX, R. LEWIS | Warminster. |
| WILLETT, GEORGE G. D. | Keynsham, Somersetshire. |
| *WILLIAMS, E. C., M.B., B.C., B.A. Cantab. | 9 Whatley Road, Clifton. |
| WILLIAMS, H. EGERTON | The Mount, Newport, Mon. |
| *WILLIAMS, LIONEL H. | Thornbury, Gloucestershire. |
| *WILLIAMS, P. WATSON, M.D. Lond. | 4 Clifton Park, Clifton. |
| WILLIAMS, PETER | Ferryside, Carmarthenshire. |
| *WILLIAMS, W. ROGER | Beaufort House, Clifton Down, Clifton. |
| WILLIS, W. M. | 24 Park Street, Nottingham. |
| *WILLS, W. K., M.B., B.C. Camb. | 59 Apsley Road, Clifton. |
| WILSON, E. T., M.D. Oxon. | Westol, Cheltenham. |
| *WINDSOR-AUBREY, H. W. | Coronation Road, Bristol. |
| *WINTLE, COLSTON | 24 Gloucester Road, Bristol. |
| WOOD, C. R., M.A., M.D. Durh. | Bridge House, Frome. |
| WOODHEAD, G. SIMS, M.D., C.M. Ed. | Pathological Laboratory, New Museum, Cambridge. |
| WOOLLCOMBE, WALTER L. | 16 The Crescent, Plymouth. |
| *YOUNG, J., M.D., C.M. Glasg. | Clouds Hill House, St. George, Bristol. |

